# Enhancing Control of *Leishmania infantum* Infection: A Multi-Epitope Nanovaccine for Durable T-Cell Immunity

**DOI:** 10.3390/ani14040605

**Published:** 2024-02-13

**Authors:** Clara Hurtado-Morillas, Abel Martínez-Rodrigo, José A. Orden, Laura de Urbina-Fuentes, Alicia Mas, Gustavo Domínguez-Bernal

**Affiliations:** 1 INMIVET, Animal Health Department, School of Veterinary Medicine, Complutense University of Madrid, 28040 Madrid, Spain; clarahur@ucm.es (C.H.-M.);; 2 INMIVET, Animal Science Department, School of Veterinary Medicine, Complutense University of Madrid, 28040 Madrid, Spain; 3 Centro de Investigación en Sanidad Animal, Instituto Nacional de Investigación y Tecnología Agraria y Alimentaria, Consejo Superior de Investigaciones Científicas (CISA-INIA-CSIC), 28130 Madrid, Spain

**Keywords:** canine leishmaniosis, PLGA nanoparticles, multi-epitope peptide, nanovaccine, LetiFend^®^, *Leishmania infantum*

## Abstract

**Simple Summary:**

Canine leishmaniosis is a potentially fatal disease in dogs caused by the *Leishmania* parasite. Vaccination seems to be the safest, most cost-effective, and long-lasting control strategy. Currently, there is still no vaccine that totally guarantees complete protection against *Leishmania* infection. Here, we designed and evaluated the effectiveness of a nanovaccine against *Leishmania infantum* infection in the murine model. This vaccine strategy, consisting of the HisDTC peptide encapsulated in polymeric nanoparticles, induced in vaccinated groups a lower parasite load in comparison to the control groups, which was correlated with the induction of a cellular immune response profile against *Leishmania infantum* measured throughout different cytokines, antibodies titers, and memory T cells. These results provide evidence that the HisDTC peptide encapsulated in polymeric nanoparticles is a potential vaccine strategy against canine leishmaniosis.

**Abstract:**

Canine leishmaniosis (CanL) is a growing health problem for which vaccination is a crucial tool for the control of disease. The successful development of an effective vaccine against this disease relies on eliciting a robust and enduring T-cell immune response involving the activation of CD4^+^ Th1 and CD8^+^ T-cells. This study aimed to evaluate the immunogenicity and prophylactic efficacy of a novel nanovaccine comprising a multi-epitope peptide, known as HisDTC, encapsulated in PLGA nanoparticles against *Leishmania infantum* infection in the murine model. The encapsulation strategy was designed to enhance antigen loading and sustain release, ensuring prolonged exposure to the immune system. Our results showed that mice immunized with PLGA-encapsulated HisDTC exhibited a significant reduction in the parasite load in the liver and spleen over both short and long-term duration. This reduction was associated with a cellular immune profile marked by elevated levels of pro-inflammatory cytokines, such as IFN-γ, and the generation of memory T cells. In conclusion, the current study establishes that PLGA-encapsulated HisDTC can promote effective and long-lasting T-cell responses against *L. infantum* in the murine model. These findings underscore the potential utility of multi-epitope vaccines, in conjunction with appropriate delivery systems, as an alternative strategy for CanL control.

## 1. Introduction

Canine leishmaniosis (CanL), a life-threatening vector-borne zoonotic disease caused by the protozoa of the genus *Leishmania*, is a significant health risk to both dogs and humans in endemic regions worldwide. This disease is present in all continents except Oceania, and it is endemic in more than fifty countries in the Mediterranean Basin, South America, and Central and Southwestern Asia [1]. Far from disease control, the incidence of CanL in non-autochthonous countries shas increased in recent years due to globalization, among other causes [2].

Among the twelve species described as capable of infecting dogs, *Leishmania (L.) infantum* is the principal factor responsible for CanL and the most widely distributed [3]. Dogs, beyond enduring the potential severity and fatality of this disease, constitute the principal reservoir for *L. infantum* in relation to human infections [1]. *L. infantum*, the etiological agent of visceral leishmaniosis (VL), represents the most severe form of this disease in humans, ranking second in mortality and fourth in morbidity among tropical diseases, with 50,000 to 90,000 cases annually [4]. Consequently, leishmaniosis emerges not only as an important disease in veterinary medicine but also as a global public health challenge for which effective control tools must be developed.

CanL’s control is, however, challenging primarily due to the high cost, limited efficacy, and potential toxicity associated with available treatments [5,6]. Compounding this issue, there are currently no effective measures to prevent human infection, which makes it even more important to design and implement adequate control measures targeting canids. Vaccination has emerged as a crucial, safe, cost-effective, and long-lasting strategy for controlling leishmaniosis. While five vaccines have been licensed against CanL, only the following two are currently commercially available: LetiFend^®^ (LETI Pharma, S.L.U., Spain) in Europe, composed of a chimera protein (protein Q) formed by five antigenic fragments from four different *L. infantum* ribosomal proteins LiP2a, LiP2b, and LiP0 and the histone H2A, and Leish-Tec^®^ (Hertape, Brazil) in Brazil, which consists of a recombinant protein A2 from *L. donovani* with saponin as a vaccine adjuvant. A third recent vaccine was approved by the European Medicines Agency (EMA) for commercialization [7]. Despite these advancements, uncertainties persist regarding their efficacy. Although these vaccines may mitigate the risk of clinical signs and disease progression, they do not guarantee complete protection against the infection establishment, and infected dogs may still serve as potential reservoirs for the parasite [8,9]. Consequently, there is a pressing need for further studies, which are necessary to develop more effective strategies capable of preventing infection and controlling the spread of this disease among both canids and humans.

Peptide vaccines have become a focal point in vaccine development because of their easy and rapid manufacture, along with their inherent stability and safety [10]. While peptide-based vaccines can elicit a specific immune response against pathogens, their immunogenic potential may be limited, necessitating the use of adjuvants, specific delivery systems, or multiple doses to enhance their immunogenicity [10]. In this context, the utilization of Toll-like receptor ligands (TLRLs) as adjuvants has shown promising results as they can promote the initiation of a robust innate immune response during the early stages of *Leishmania* infection, paving the way for the development of an effective cell-mediated immune response [11,12]. Several licensed vaccines currently incorporate TLRLs as adjuvants, such as TLRL-4 [13,14] and TLRL-9 [15]. Ongoing studies are exploring other Toll-like receptor agonists, such as TLRL-2 (Pam3CSK4) and TLRL-3 (Polyinosinic-polycytidylic acid (poly(I:C)) as potential vaccine adjuvants against leishmaniosis [16,17,18].

In this context, in parallel to the use of adjuvants, nanotechnology has introduced innovative possibilities for designing vaccine delivery systems against various infectious diseases, including leishmaniosis [19,20,21]. Among different nanoparticles (NPs), poly(lactic-*co*-glycolic) acid (PLGA) nanoparticles, approved by the EMA, have gained considerable attention as carriers for antigen delivery in vaccine development due to their properties. The biodegradable and biocompatible nature of PLGA allows for the safe administration and controlled release of encapsulated antigens. Additionally, PLGA nanoparticles protect antigens from degradation, enhance antigen uptake through antigen-presenting cells, and facilitate co-encapsulation with adjuvants, thereby enhancing immune responses [22].

This study specifically explores the use of the HisDTC multi-epitope peptide vaccine delivered by PLGA NP in combination with TLRLs as adjuvants. This peptide has already been described to induce a specific cellular immune response in vaccinated mice that effectively controls parasite multiplication against *L. infantum* [23]. To enhance the long-term efficacy of the HisDTC-induced immune response against *L. infantum* infection in BALB/c mice, this study evaluates the co-encapsulation of the multi-epitope peptide with TLRL-2 and TLRL-3 as immunomodulators with PLGA nanoparticles. For this purpose, we analyzed the parasite load in target organs and characterized the specific immune response generated against *L. infantum* through this vaccine strategy in the murine model of VL.

The use of the murine model serves as a crucial initial step towards evaluating the vaccine’s efficacy. Furthermore, this study highlights the promising role of nanotechnology-based vaccine delivery systems in overcoming challenges associated with vaccine development for complex infectious diseases such as leishmaniosis.

## 2. Materials and Methods

### 2.1. Mice, Parasites, and Soluble Leishmania Antigen Production

Studies were performed on 6–8-week-old female BALB/c mice (Janvier-Labs, Laval, France), which were housed in the Animal Facility of the Complutense University of Madrid under specific pathogen-free conditions. The study was approved (reference: PROEX 211/18) by the Animal Welfare Committee of the Community of Madrid, Spain. Animal care and procedures followed Spanish and EU legislation (Law 32/2007, R.D. 53/2013, and Council Directive 2010/63/EU).

*Leishmania infantum* BCN150 zymodeme MON-1 (M/CAN/ES/96/BCN150) promastigotes were cultured, as reported previously, at 26 °C in Schneider’s medium (Sigma-Aldrich, Saint Louis, MO, USA) supplemented with 20% of inactivated fetal bovine serum (FBS, Sigma-Aldrich, Saint Louis, MO, USA), 200 U/mL of penicillin and 200 mg/mL of streptomycin (Lonza, Basel, Switzerland) [24]. All the parasites were previously passaged in BALB/c mice to maintain their virulence [25]. The specific soluble *Leishmania* antigen (SLA) was prepared from stationary-phase *L. infantum* promastigotes as described elsewhere, and it was stored at −20 °C until use [26].

### 2.2. Vaccine Preparation

#### 2.2.1. Chimeric Multi-Epitope Peptide Formulation and TLRL Adjuvants Synthesis

The multi-epitope peptide vaccine HisDTC was previously designed, as detailed in [23]. The peptides were synthesized by GenScript Biotech (Leiden, The Netherlands) with purity ≥ 95% and stored at −20 °C until encapsulation.

TLRL-2 (Pam3CSK4) and TLRL-3 (Poly(I:C) HMW (VacciGrade™, InvivoGen, Toulouse, France) were used as adjuvants when needed.

#### 2.2.2. PLGA Nanoparticles Preparation and Characterization

PLGA-NPs containing the chimeric peptide HisDTC and/or immunomodulators TLRL-2/3, when required, were performed using the double emulsion method (w/o/w) by Nanovex Biotechnologies (Llanera, Asturias, Spain). Briefly, a solution of PLGA (lactide/glycolide 50:50 molar ratio) was prepared in dichloromethane (20 mg/mL) and mixed with 1 mL of aqueous medium containing, when needed, the corresponding amount of each active ingredient to be encapsulated (peptide HisDTC; TLRL-2; TLRL-3) forming a primary solution of water in oil. The resulting solution was then added to a second aqueous solution containing polyvinyl acetate (PVA) 2%, and the mixture was emulsified via homogenization. The final double (w/o/w) emulsion was left under constant homogenization for 18 h to allow the organic solvent to evaporate. Nanoparticles were then purified by subjecting them to three consecutive cycles of centrifugation and redispersion. The final collected PLGA formulations were dispersed in a phosphate final medium pH 7 with 3% sucrose to stabilize PLGA nanoparticles for subsequent lyophilization, which were conserved at 4 °C until use.

The particle size and polydispersity index (PDI) of the synthesized nanoparticles were characterized using the Dynamic Light Scattering (DLS) technique and the Zetasizer ZS90 (Marlvern Instruments Ltd., Marlvern, UK). Zeta potential measurements were performed at 25 °C using the M3-PALS (Mixed Measurement Mode Phase Analysis) technique and the Zetasizer ZS90 (Marlvern Instruments Ltd.).

TLRL-3 and TLRL-2 concentrations were determined using the Quant-iT™ OliGreen ssDNA Reagent Assay Kit (Invitrogen, Molecular Probes, Eugene, OR, USA) and OPA (Fluoraldehyde) kit (Thermo Scientific, Waltham, MA, USA), respectively. The concentration of HisDTC was determined using the BCA kit (Thermo Scientific, Waltham, MA, USA). A Synergy™ HTX Multi-Mode Microplate Reader (Agilent Technologies, Thermo Scientific, Waltham, MA, USA) was used for fluorescence and absorbance measurements. All measurements were conducted using aqueous dispersions of PLGA-NPs prior to their lyophilization.

### 2.3. Immunization, Infection and Sacrifice of Mice

BALB/c mice were divided into five groups (*n* = 9 per group) and immunized subcutaneously on weeks 0 and 2, with a total volume of 100 µL of sterile phosphate-buffered saline (PBS) containing the following formulations: group 1 mice were immunized with 10 µg of HisDTC, 5 µg TLRL-2 and 38 µg of TLRL-3 encapsulated in PLGA-NPs (HisDTC-TLRLs-PLGA group); group 2 mice were immunized with 10 µg of HisDTC encapsulated (HisDTC-PLGA group); group 3 mice were and with 10 µg of protein Q (LetiFend group); groups 4 and 5 served as control mice which received 5 µg of TLRL-2 and 38 µg of TLRL-3 encapsulated in PLGA nanoparticles (TLRLs-PLGA group) and PLGA empty nanoparticles (PLGA group), respectively.

Animals were stratified into two analyses. Firstly, to assess the short-term immunoprophylactic capacity of the formulations on week 6, five mice per group were infected intravenously with 5 × 10^5^ stationary-phase *L. infantum* promastigotes (short-term evaluation). Lately, with the aim of studying whether the effectiveness of the vaccination was prolonged in the long term, four mice per group were infected intravenously with 5 × 10^5^ stationary-phase *L. infantum* promastigotes on week fourteen (long-term evaluation). In both cases, seven weeks post-infection, the animals were sacrificed, and serum samples, the liver, and spleen were collected to perform parasitological and immunological analyses.

### 2.4. Evaluation of Delayed-Type Hypersensitivity (DTH)

The cell-mediated immune response was examined by assessing the DTH reaction. Mice received an intradermal injection of 50 µg of SLA in a 35 µL volume of sterile PBS into the right footpad two days prior to infection for short and long-term evaluation. DTH response was assessed by measuring the difference in footpad swelling 48 h after SLA injection compared to the left footpad that received a control PBS injection, using a digital caliper (Sigma-Aldrich, Saint Louis, MO, USA).

### 2.5. Quantification of Parasite Burden Using Limited Dilution Assay

Parasite burdens in the liver and spleen were quantified using the limited dilution assay [27]. Briefly, the spleen and a portion of the liver that had been previously weighed were homogenized in 4 mL of Schneider’s Insect Medium supplemented with 20% FBS. Subsequently, tissue homogenates were serially diluted (4-fold) in microtiter culture plates (Corning Costar, Thermo Scientific, Waltham, MA, USA) and incubated in quadruplicate at 26 °C. After eight days, the presence of motile promastigotes was examined using an inverted microscope. The parasite load was determined as the last positive dilution containing at least one viable parasite; it was expressed as the parasite number per gram of tissue, in the case of the liver, or per tissue, in the case of the spleen.

### 2.6. Isolation and Co-Culture System of Bone Marrow-Derived Murine Dendritic Cells (BMDCs)

Ten days before sacrifice, bone marrow stem cell progenitors were harvested from the femurs and tibias of naïve BALB/c mice (*n* = 5) and cultured in RPMI 1640 with L-glutamine and 25 mM of HEPES (Sigma-Aldrich, Saint Louis, MO, USA), supplemented with 10% FBS and a mixture of antibiotics (100 U/mL penicillin, 100 mg/mL streptomycin, Lonza) in the presence of 20 ng/mL of the murine granulocyte-macrophage colony-stimulating factor (GM-CSF; BioLegend^®^, San Diego, CA, USA), modified from the protocol previously described [28]. Fresh medium containing GM-CSF was added to the cultures every 3 days. On day 6, cells were harvested, resuspended in RPMI medium with a freshly added growth factor, and cultured at 37 °C with 5% CO_2_. Finally, on day 9, non-adherent naïve BMDCs were seeded in 24-well plates (5 × 10^5^ cells/mL) and used for subsequent experiments. 

### 2.7. Extracellular Cytokine Profile and Arginine Metabolism Elicited by Ex Vivo Co-Cultured Splenocytes Stimulations

To quantify cytokine production in immunized and infected mice, BMDCs obtained from naïve mice were plated (5 × 10^5^ cells/mL) into 24-well plates and either stimulated or not with 25 µg/mL of SLA overnight. Following this, splenocytes obtained from immunized and infected mice were co-cultured at a ratio of 1:5 (BMDCs/splenocytes) at 37 °C and 5% CO_2_. After 96 h, supernatants were collected and stored at –80 °C until processing. The levels of IFN-γ (ELISA MAX^TM^ Deluxe Set Mouse, BioLegend, San Diego, CA, USA), TNF-α (Duoset ELISA kit, Development System R&D, Abingdon, UK), and IL-10 (BD OptEIA kit, Bioscience, San Diego, CA, USA) were quantified using commercially available ELISA kits following the instructions provided by the manufacturers.

In parallel, L-arginine metabolism was evaluated by quantifying nitric oxide (NO) production and arginase activity using the same experimental set. After 96 h, the co-culture of naïve BMDCs with splenocytes from the animals of different groups in the presence of 25 µg/mL of SLA at 37 °C and 5% CO_2_. NO production was measured indirectly from the concentration of nitrites in supernatants using the Griess reaction as described [29]. Intracellular arginase activity was estimated using an enzymatic-colorimetric assay method, which detects urea production [30].

### 2.8. Flow Cytometric Characterization and Immunophenotyping of Cellular Immune Response

Seven weeks post-infection, spleen samples were collected to assess cytokine-producing T lymphocytes and characterize memory T cell subpopulations triggered by vaccination. Spleen samples from immunized and infected animals were macerated, the red blood cells lysed, and the isolated spleen cells were stimulated ex vivo with SLA (25 μg/mL) at 37 °C with 5% CO_2_ for 12 h to characterize T lymphocyte subpopulations or for 48 h to assess intracellular cytokine staining. These simulations were performed in polypropylene tubes (Falcon, BD Pharmingen, San Diego, CA, USA) at a concentration of 1 × 10^6^ cells/mL. The non-stimulated culture received only the culture medium.

Dead cells were excluded using the LIVE/DEAD Zombie RED Fixable Viability Kit (BioLegend, San Diego, CA, USA). The events (200,000 live cells) were acquired on a CytoFLEX S flow cytometer (Beckman Coulter, Life Science, Brea, CA, USA), and the CytExpert™ 2.4 software package (Beckman Coulter, Life Science, Brea, CA, USA) was utilized for data analysis.

#### 2.8.1. Intracellular Cytokine Production

Upon stimulation and prior to harvest, isolated spleen cells were pre-incubated with brefeldin A (BioLegend, San Diego, CA, USA) for 4 h at 37 °C. To block non-specific antibody binding, the TruStain FcX™ antibody (Clone 96, BioLegend, San Diego, CA, USA) was then added. Subsequently, splenocytes were incubated with Zombie RED (Fixable Viability Kit, BioLegend, San Diego, CA, USA) for 10 min at room temperature, washed and immunostained with anti-CD4 FITC (clone GK1.5, BioLegend) (diluted 1:800) and anti-CD8 PE (clone QA17A07, BioLegend) (diluted 1:800) fluorophore-labeled antibodies. After two washes with PBS containing 2% FCS, cells were fixed and permeabilized using Cyto-FastTM Fix/Perm Solution (BioLegend) according to the manufacturer’s protocol. Then, cells were stained with anti-IFN-γ PerCP (clone XMG1.2, BioLegend) and anti-IL-10 PCy7 (clone JES5-16E3, BioLegend) at room temperature for 30 min, washed twice, and analyzed. The production of intracellular cytokines was expressed as percentages, representing the ratio of the percentage of IFN-γ or IL-10-producing-T cells in SLA-stimulated cultures.

#### 2.8.2. Specific T Lymphocyte Subsets

For immunophenotyping T lymphocyte memory subsets, the TruStain FcX™ antibody (Clone 96, BioLegend) was added to block the non-specific binding of antibodies. Subsequently, splenocytes were incubated with Zombie RED for 10 min at room temperature. Finally, cells were stained with the following fluorophore-labeled anti-mouse monoclonal antibodies: anti-CD4 FITC (clone GK1.5, BioLegend), anti-CD8 PCy7 (clone QA17A07, BioLegend), anti-CD44 PE (clone IM7, BioLegend) and anti-CD62L PerCP (clone MEL-14, BioLegend) for 20 min at 4 °C in the dark. The T lymphocyte subsets were characterized by the percentage values obtained from the SLA-stimulated culture.

### 2.9. Leishmania-Specific IgG1 and IgG2a Antibody Titration

Sera samples from immunized and infected mice were used for the titration of the IgG isotype antibody using the ELISA assay as previously described [31]. Briefly, mice serum was serially two-fold-diluted starting from 1:400 for 90 min in an ELISA 96-well microtiter plate (Nunc Immunoplate MaxiSorp, Thermo Scientific, Waltham, MA, USA) previously coated with 6 µg/mL of specific SLA antigens. Next, the IgG isotype goat anti-mouse antibody conjugated with peroxidase (HRP) (Southern Biotech, Birmingham, AL, USA) was added for 1 h. Finally, the reaction was detected using the TMB substrate (Thermo Scientific, Waltham, MA, USA). The optical density was measured at 450 nm, and the reciprocal end-point titer was determined as the highest serum dilution, providing an absorbance three times higher than the negative control (serum from uninfected and non-immunized mice).

### 2.10. Statistical Analysis

Statistical analyses were performed using GraphPad Prism software (version 8.3 for Windows, San Diego, CA, USA). First, the Shapiro–Wilk normality test was performed to assess the normality distribution of quantitative variables. Then, one or two-way ANOVA with multiple comparisons of Tukey’s post hoc test was conducted to determine which means from the independent groups were significantly different. The antibody response of animals was analyzed using Kruskal–Wallis’s test, as these data did not follow a normal distribution. Statistical significance in all models was considered for *p*-values ≤ 0.05.

## 3. Results

### 3.1. PLGA-Encapsulated HisDTC Induces a Robust and Durable DTH Response

The PLGA polymeric nanoparticles prepared using the w/o/w solvent evaporation method had a regular morphology and a smooth surface. The particle sizes used for this study ranged from 243 to 287 nm, presenting a PDI of 0.09 ± 0.02. The Zeta potential of these formulations used in the present work had an average of −29 mV.

**Figure 1:**
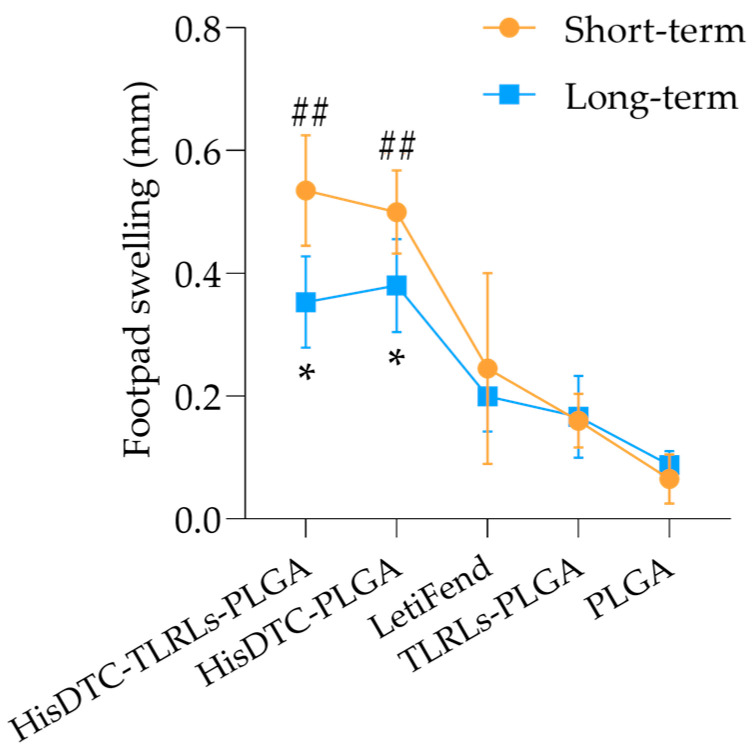
Delayed type of hypersensitivity response in immunized mice. BALB/c mice were immunized twice. Prior to infection, four weeks after the last immunization(short-term), and fourteen weeks post-immunization (long-term), DTH was assessed. After 48 h of SLA injection in the right footpad, inflammation was measured, and the difference with the control left footpad injected with PBS was calculated. Data are represented as the mean ± SEM. Statistical analysis was performed with one-way ANOVA followed by Turkey’s post hoc test. Hashes indicate significant differences (##; *p* < 0.01) between immunized and control groups (TLRLs-PLGA and PLGA). Asterisks indicate differences (*; *p* < 0.05) between the immunized and PLGA control group.

PLGA-encapsulated HisDTC vaccine formulations were evaluated for their ability to induce a DTH response after the immunization of BALB/c mice. These formulations included the multi-epitope peptide HisDTC with or without TLRLs (HisDTC-TLRLs-PLGA and HisDTC-PLGA groups, respectively). Additionally, the LetiFend^®^ vaccine (LetiFend group) was used as a reference, enabling a comparative assessment of the vaccine’s effectiveness against CanL. Control groups included the administration of encapsulated TLRL-2/3 (TLRLs-PLGA group) and empty PLGA nanoparticles (PLGA group). All vaccine strategies were administered twice at 14-day intervals via the subcutaneous route. The DTH reaction was studied as an indicator of a specific cell-mediated immune response against *Leishmania* induced by vaccination prior to infection. A short-term (four weeks after the last immunization) evaluation of the DTH response revealed that mice immunized with HisDTC (both HisDTC-TLRLs-PLGA and HisDTC-PLGA groups) exhibited significantly (*p* < 0.01) higher swelling rates compared to the rest of the groups (Figure 1). In contrast, mice vaccinated with LetiFend^®^ and control groups (TLRLs-PLGA and PLGA) showed similar magnitudes of DTH response, with no statistical differences between them.

Interestingly, for the long-term evaluation (fourteen weeks after the last immunization), HisDTC-vaccinated mice (with and without adjuvants) displayed milder DTH responses compared to the short-term evaluation but still demonstrated the most pronounced induration. They were the only groups that presented statistically significant (*p* < 0.05) differences compared to the PLGA control group (Figure 1).

### 3.2. Immunization with HisDTC-Based PLGA Nanoformulations Confer Short and Long-Lasting Protection against L. infantum Infection

To assess the efficacy and durability of protection conferred by the HisDTC peptide encapsulated in PLGA nanoparticles against *L. infantum* infection, immunized mice were infected with stationary phase *L. infantum* promastigotes either four weeks (short-term evaluation) or fourteen weeks (long-term evaluation) after the last immunization. Subsequently, seven weeks post-infection, the animals were euthanized, and parasite load quantification was performed in target organs using a limiting dilution assay.

**Figure 2:**
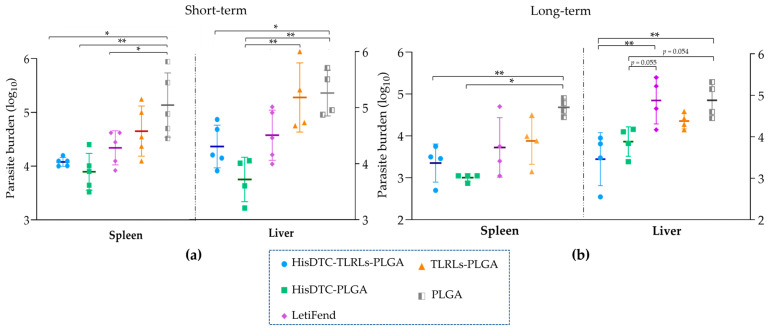
Splenic and liver parasite burden in immunized and infected BALB/c mice. BALB/c mice were infected with stationary phase *L. infantum* promastigotes four weeks after the last immunization in short-term vaccinated and evaluated mice (**a**) or fourteen weeks after the last immunization in long-term evaluated mice (**b**). Seven weeks post-infection, mice were sacrificed, and parasite burden was calculated by limiting the dilution assay in the spleen and liver. Data are represented as the mean ± SD. Statistical analysis was performed with two-way ANOVA followed by Tukey’s test for multiple comparisons. Asterisks (*) indicate statistically significant differences (*; *p* < 0.05, **; *p* < 0.01) between groups.

For the short-term evaluation (Figure 2a), the control groups (PLGA and TLRLs-PLGA) exhibited the highest parasite loads in both the spleen and liver organs. Conversely, mice immunized with HisDTC, w/o TLRLs (HisDTC-TLRLs-PLGA and HisDTC-PLGA groups), as well as LetiFend-vaccinated animals, displayed a lower parasite load compared to both control groups. This decrease was statistically significant in mice immunized with the HisDTC peptide alone (*p* < 0.05). Specifically, of these two HisDTC immunized groups, HisDTC without immunomodulators was the most effective at controlling *L. infantum* multiplication, presenting the statistically significant lowest parasite burdens among all groups (*p* < 0.01).

Similarly, for the long-term evaluation (Figure 2b), both HisDTC-PLGA and HisDTC-TLRLs-PLGA groups were the only ones that maintained durable protection, exhibiting statistically significant (*p* < 0.05) lower parasite loads compared to the PLGA control group in both target organs and the LetiFend group in the liver. Surprisingly, the analysis revealed that the parasite burden displayed a similar load in LetiFend and both control groups (TLRLs-PLGA and PLGA) when long-term protection was evaluated.

### 3.3. Multipeptide HisDTC Elicits a Robust and Durable Antigen-Specific T-Cell Immune Response

**Figure 3:**
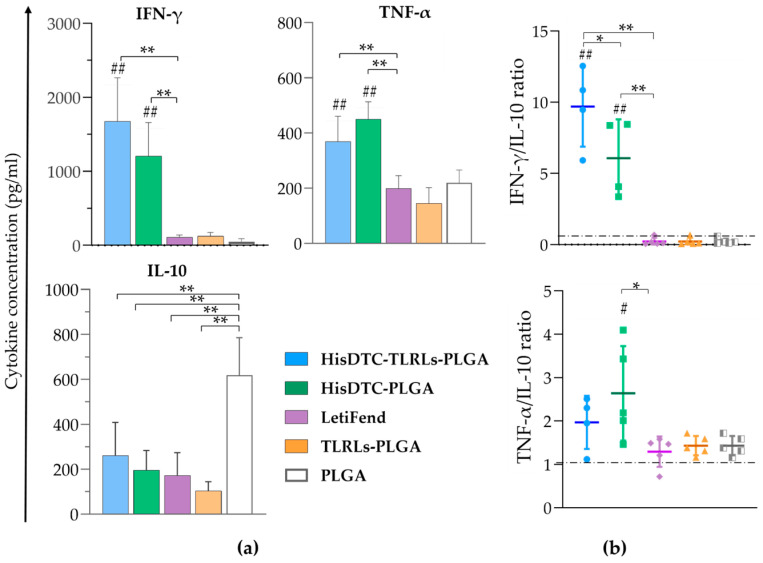
Cytokine production in splenocytes from immunized mice for the short-term evaluation. Spleen cells from short-term-evaluated vaccinated and infected animals were collected and co-cultured with naïve BMDCs stimulated with SLA. Levels of IFN-γ, TNF-α, and IL-10 were assessed using ELISA in culture supernatants. Panel (**a**) shows the mean ± SD of cytokine concentrations (pg/mL). Panel (**b**) shows the IFN-γ/IL-10 and TNF-α/IL-10 ratios. Statistical analysis was performed with one-way ANOVA followed by Tukey’s test for multiple comparisons. Hashes indicate significant differences (#; *p* < 0.05; ##, *p* < 0.01) between immunized and control groups (TLRLs-PLGA and PLGA). Asterisks indicate statistically significant differences (*; *p* < 0.05, **; *p* < 0.01) between groups.

The ability of PLGA-NP formulations to induce and activate a protective antigenic specific cellular immune response against *L. infantum* infection in vaccinated mice was assessed by quantifying Th1 (IFN-γ and TNF-α) and Th2 (IL-10) characteristic cytokine profiles. For this purpose, cytokine concentrations were measured using ELISA in co-culture supernatants of splenocytes from immunized and infected mice with SLA-stimulated naïve BMDCs for 96 h. For the short-term evaluation (Figure 3), all HisDTC-vaccinated mice exhibited a significant increase (*p* < 0.01) in pro-inflammatory IFN-γ and TNF-α cytokine production compared to other groups (Figure 3a). Notably, HisDTC-immunized mice showed a significant reduction (*p* < 0.01) in anti-inflammatory IL-10 cytokine production compared with the PLGA control group. In contrast, the LetiFend vaccinated group did not show significant differences in proinflammatory cytokine production when compared to the control groups (Figure 3a).

To assess the Th1/Th2 balance and overall immune response, cytokine ratios IFN-γ/IL-10 and TNF-α/IL-10 were calculated. HisDTC-immunized groups (HisDTC-TLRLs-PLGA and HisDTC-PLGA) exhibited the highest IFN-γ/IL-10 ratios (*p* < 0.01) compared to the rest of the groups (Figure 3b). Additionally, mice immunized with HisDTC without TLRs as adjuvants showed statistically significant differences (*p* < 0.05) in the TNF-α/IL-10 ratio compared to TLRLs-PLGA and PLGA control groups (Figure 3b). Despite the LetiFend and TLRLs-PLGA groups showing a statistically significant lower IL-10 production (*p* < 0.01) compared to the PLGA group (Figure 3a), no differences in cytokine ratios were observed among them (Figure 3b). These results pointed to the predominance of a Th1 immune response in the Th1/Th2 immunological balance, which is related to the better control of parasite multiplication in terms of the parasite load showed previously in all animals that were immunized with the HisDTC peptide encapsulated.

**Figure 4:**
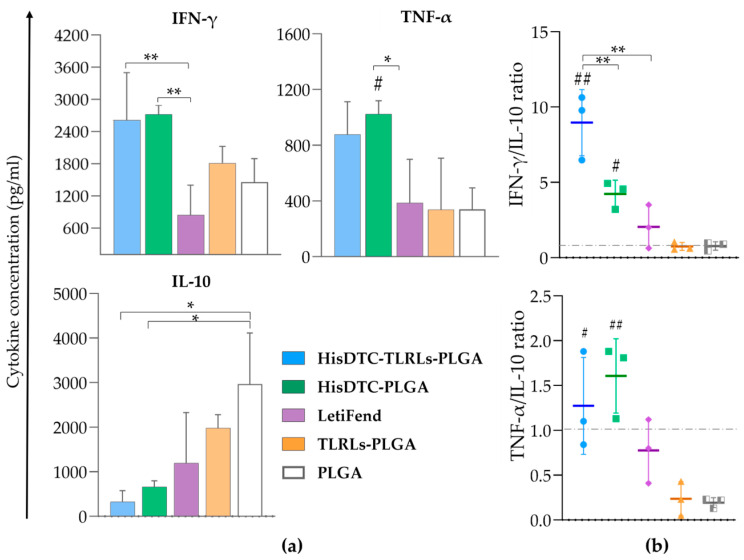
Cytokine production in splenocytes from immunized mice for long-term evaluation. Spleen cells from long-term-evaluated vaccinated and infected animals were collected and co-cultured with naïve BMDCs stimulated with SLA. Levels of IFN-γ, TNF-α, and IL-10 were assessed using ELISA in culture supernatants. Panel (**a**) shows the mean ± SD of cytokine concentrations (pg/mL). Panel (**b**) shows the IFN-γ/IL-10 and TNF-α/IL-10 ratios. Statistical analysis was performed with one-way ANOVA followed by Tukey’s test for multiple comparisons. Hashes indicate significant differences (#; *p* < 0.05; ##; *p* < 0.01) between immunized and control groups (TLRLs-PLGA and PLGA). Asterisks indicate statistically significant differences (*; *p* < 0.05, **; *p* < 0.01) between groups.

To investigate the underlying cause of the lasting protection observed in HisDTC-immunized mice for long-term evaluation, we examined their immunological responses. HisDTC-vaccinated groups consistently produced higher levels of IFN-γ and TNF-α cytokines and lower concentrations of the IL-10 cytokine in the co-cultured system compared to the other groups (Figure 4a). The IFN-γ/IL-10 and TNF-α/IL-10 ratios (Figure 4b), similar to the short-term evaluation, indicated a Th1-dominant response in mice vaccinated with the HisDTC peptide, which is associated with the effective control of *Leishmania* multiplication. These results highlight the capacity of the encapsulated multi-peptide HisDTC to promote a robust and durable Th1-dominant response, contributing to the sustained protection observed over time.

### 3.4. HisDTC Nanoformulations Induce a Robust and Long-Term Th1 Memory Response Triggered by T CD8^+^ Lymphocytes

In some parasitic infectious diseases such as leishmaniosis or malaria, the efficacy of vaccines relies not only on the balance of cytokine production but also on the activation and induction of memory subsets of CD4^+^ and CD8^+^ T lymphocytes [32]. Thus, flow cytometry assays were conducted to assess the populations of T CD4^+^ and CD8^+^ cells producing IFN-γ and IL-10 as well as their respective memory lymphocyte subpopulations. 

**Figure 5:**
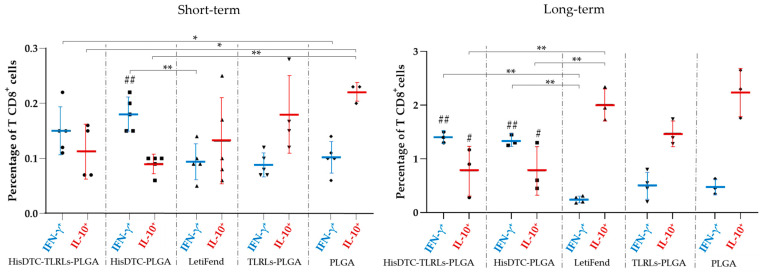
*L. infantum*-specific IFN-γ and IL-10-producing CD8^+^ T cells in immunized mice. BALB/c mice were immunized with two doses of different nanoformulations and the LetiFend^®^ vaccine. Four weeks and fourteen weeks after the last immunization, animals were infected with *L. infantum* and euthanized seven weeks post-infection. Spleen cells were collected and stimulated with SLA. After 48 h, the frequency of CD8^+^ T lymphocytes producing IFN-γ or IL-10 was assessed using the flow cytometry assay. Data show the mean ± SD of the percentage of Ag-specific CD8^+^ T cells. Statistical analysis was performed with two-way ANOVA followed by Turkey’s post hoc test. Hashes indicate significant differences (#; *p* < 0.05; ##; *p* < 0.01) between HisDTC-immunized and control groups (TLRLs-PLGA and PLGA). Asterisks indicate statistically significant differences (*; *p* < 0.05, **; *p* < 0.01) between groups.

While no significant differences were observed in cytokine-producing CD4^+^ T cells (data not shown), mice immunized with the chimera HisDTC-encapsulated w/o TLR adjuvants exhibited higher percentages of IFN-γ^+^-producing CD8^+^ T-lymphocytes compared to the rest of the groups, including the LetiFend group. Notably, statistically significant differences were observed only in the case of HisDTC nanoformulations (*p* < 0.01) for the short-term evaluation and in both HisDTC w/o adjuvant groups for the long-term evaluation (Figure 5). It is worth noting the increased presence of CD8^+^ IFN-γ-producing T cells in HisDTC-vaccinated animals for the long-term evaluation was up to three-fold times higher compared to LetiFend and control groups.

An inverse relationship between the percentage of IFN-γ^+^-producing T CD8 cells and IL-10^+^-producing T CD8^+^ cells was observed for both the short- and long-term evaluation (Figure 5). Consequently, HisDTC-vaccinated groups, characterized by the highest percentages of IFN-γ^+^ T CD8^+^ lymphocytes, exhibited fewer IL-10^+^ T CD8^+^ cells in contrast to the control and LetiFend groups. This difference was statistically significant during the long-term evaluation (*p* < 0.01). These findings highlight the ability of HisDTC encapsulated in PLGA w/o TLR as an adjuvant to promote a Th1-polarized response through the secretion of IFN-γ over IL-10, triggered by T CD8^+^ lymphocytes against *L. infantum* infection in contrast to the control groups and even the LetiFend group.

**Figure 6:**
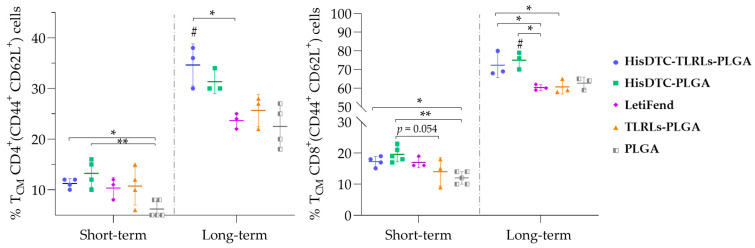
Frequencies of central memory T lymphocytes in immunized and infected mice. Spleen cells were previously stimulated for 12 h with SLA and analyzed via flow cytometry for immunophenotyping T cell memory based on CD62L and CD44 expression. The CD62L^+^ CD44^+^ central memory (T_CM_) CD4 and CD8 T lymphocyte frequencies are depicted. Data represent the mean ± SD. Statistical analysis was performed with one-way ANOVA followed by Turkey’s post hoc test. Hashes indicate significant differences (#; *p* < 0.05) between HisDTC-immunized and control groups (TLRLs-PLGA and PLGA). Asterisks indicate statistically significant differences (*; *p* < 0.05, **; *p* < 0.01) between groups.

In addition to cytokine-specific production, the induction of memory lymphocytes due to vaccination was also evaluated. The results revealed that, for the short-term evaluation, chimera HisDTC encapsulated w/o TRL as this adjuvant induced the highest percentages of central memory lymphocytes in both CD4^+^ and CD8^+^ cell subpopulations (CD4^+^/CD8^+^ CD44^+^ CD62L^+^) in infected mice when compared to the other groups. This was statistically significant (*p* < 0.05) when compared to the PLGA control group (Figure 6).

To determine the long-lasting nature of these memory T cells and their implication in long-term protection against *L. infantum*, these memory cells were re-evaluated twenty-one weeks after the last immunization (long-term evaluation). Interestingly, HisDTC-TLRLs-PLGA and HisDTC-PLGA presented higher percentages of CD4^+^ and CD8^+^ memory T cells. It is noteworthy that control groups, as well as the LetiFend group, exhibited the same percentages in both CD4^+^ and CD8^+^ T cell subsets for the short- and long-term evaluation. These results may be correlated with the long-lasting capacity of HisDTC nanoformulations observed in vaccinated animals to inhibit *L. infantum* multiplications in target organs.

### 3.5. Vaccination with HisDTC Elicited Antigen-Specific Humoral Immune Responses after L. infantum Infection

**Figure 7:**
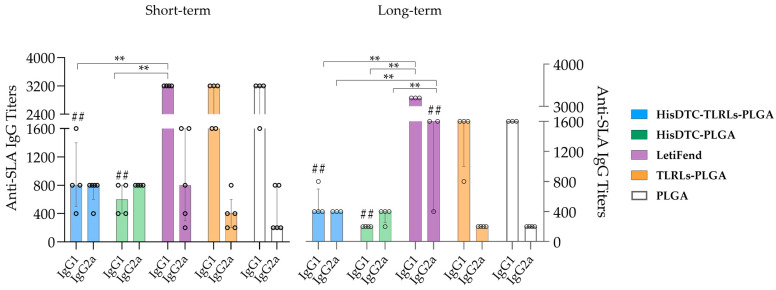
*Leishmania infantum*-specific IgG1 and IgG2a subclass antibody titers in immunized and infected mice. Serum samples were collected from all mice groups prior to sacrifice. Anti-SLA-specific IgG responses were measured via ELISA using an SLA-coated ELISA plate and HRP-conjugated antisera to detect IgG1 and IgG2a isotype levels in short- and long-term evaluations. Data are represented as the median and interquartile range. Hashes (##; *p* < 0.01; Kruskal–Wallis’s test) denote a significant difference between median IgG levels in the immunized (HisDTC-TLRLs-PLGA, HisDTC-PLGA, and LetiFend) and control (TLRLs-PLGA and PLGA) groups. Asterisks denote significant differences between HisDTC- and LetiFend-immunized mice groups (**; *p* < 0.01; Kruskal–Wallis´s test).

After the successful control of parasite multiplication in vaccinated animals, we investigated the immune correlates of protection associated with immunoglobulin (Ig) production. Since the production of IgG2a antibodies is typically associated with a Th1-type immune response, whereas IgG1 is associated with a Th2-type immune response, we quantified the presence of anti-SLA antibodies for these two IgG isotypes in sera samples from immunized and infected mice seven weeks post-infection. As expected, for the short-term evaluation, the median anti-SLA IgG1 titer obtained by control groups (TLRs-PLGA and PLGA) was significantly higher (*p* < 0.01) than that obtained via immunization with encapsulated HisDTC (with and without adjuvants) (Figure 7). Interestingly, mice immunized with the LetiFend^®^ vaccine exhibited a similar median IgG1 titer as the control groups and a statistically greater titer (*p* < 0.01) than mice immunized with HisDTC-encapsulated w/o adjuvants (Figure 7). Conversely, although no statistically significant differences were observed, the titer of IgG2a antibodies was higher in mice immunized with HisDTC and adjuvants (1:800), HisDTC alone (1:800), and LetiFend (1:800) compared to the control TLRLs-PLGA (1:400) and PLGA (1:200) groups.

Consistent with previous results, antibody levels showed the same pattern for the long-term evaluation, with lower IgG1 titers (*p* < 0.01) in mice immunized with HisDTC compared to the LetiFend and control groups (Figure 7). The only notable difference observed was that LetiFend when evaluating long-term immunity, induced a statistically significant higher production (*p* < 0.01) of IgG2a antibodies compared to the rest of the groups. However, the only group that demonstrated the predominance of the IgG2a antibody over IgG1 was the group of mice vaccinated with HisDTC chimera without adjuvants, pointing to a greater capacity to effectively control the infection.

### 3.6. Immunization with Encapsulated HisDTC Induces a Protective L-Arginine Metabolic Pathway against Parasite Multiplication

The metabolism of the amino acid L-arginine, involving the enzymes nitric oxide synthetase (iNOS) and arginase, has significant implications for *Leishmania* infection in mice. iNOS generates NO from L-arginine, a potent leishmanicidal molecule that aids in parasite control. However, arginase synthesizes ornithine and polyamine products, promoting parasite survival [33]. Consequently, the balance of arginase activity and NO production is pivotal in determining resistance or susceptibility profiles to *Leishmania* infection. After 96 h of the co-culture of splenocytes from immunized animals and naïve BMDCs stimulated with SLA, these two enzymatic parameters were quantified.

**Figure 8:**
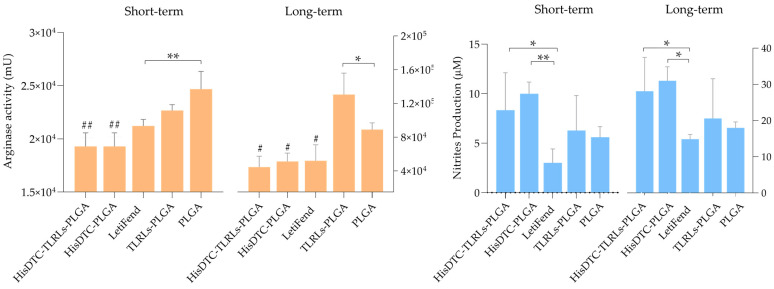
Arginine metabolism in naïve BMDCs co-cultured with splenocytes from vaccinated and infected mice. (**a**) The intracellular arginase activity (Mu) was estimated using the enzymatic-colorimetric assay method; (**b**) NO production was measured indirectly from the concentration of nitrites in supernatants using the Griess reaction. Data are represented as the mean ± SD. Hashes (one-way ANOVA: #; *p* < 0.05; ##; *p* < 0.01) denote a significant difference between mean levels in the immunized (HisDTC-TLRLs-PLGA, HisDTC-PLGA, and LetiFend) and control (TLRLs-PLGA and PLGA) groups. Asterisks (one-way ANOVA: *; *p* < 0.05; **; *p* < 0.01) denote significant differences between groups.

The results obtained reveal that mice vaccinated with encapsulated peptide HisDTC, w/o TLRLs both in short- and long-term evaluations, exhibited higher NO production and lower arginase levels when compared to the control groups (TLRLs-PLGA and PLGA). Notably, this difference was statistically significant only in data obtained from the arginase activity measurement (Figure 8). These findings indicate that encapsulated HisDTC has the capability to induce, in vaccinated animals, L-arginine metabolism that favors the control of disease progression.

## 4. Discussion

CanL, caused by the protozoa *Leishmania infantum* species, poses significant clinical, epidemiological, and zoonotic challenges. The development of an effective preventive canine vaccine is crucial for its control [1]. Currently, commercially available vaccines such as Leishtec^®^ (Hertape, Brazil) in Brazil and LetiFend^®^ (LETI Pharma, S.L.U., Spain) in Europe exist but demonstrate limited efficacy against *L. infantum* infection in terms of disease progression and transmission [8]. These vaccines exhibit suboptimal efficacy against *L. infantum* infection, both in terms of disease progression and transmission, which is potentially attributed to the formulation of recombinant protein vaccines. Second-generation vaccines of this nature, while effective for other diseases, may encounter concerns related to low immunogenicity when administered without an adjuvant [10]. The successful development of an effective vaccine against leishmaniosis relies on the establishment of a specific and durable T-cell immune response, primarily driven by the activation of memory and effector T CD4^+^ and CD8^+^ lymphocytes [34,35]. To address this challenge, there is a growing exploration of peptide vaccines delivered through various platforms and adjuvants. [36,37]. Along this line, our group innovated by designing and developing a chimeric multi-epitope peptide named HisDTC. This peptide, whether administered alone or in combination with saponin, has demonstrated its capacity to induce a specific T-cell response, aiding immunized animals in controlling *L. infantum* multiplication in murine models [23,38]. Nevertheless, the optimization of this strategy is imperative to enhance the control of disease progression and ascertain the durability of the induced immunity.

In the present study, we conducted a comprehensive assessment of the short- and long-term immunoprophylactic efficacy of the multi-epitope peptide HisDTC. We explored its potential in the presence and absence of TLRL-2 and TLRL-3 and recognized immunomodulators co-encapsulated in PLGA polymeric nanoparticles in BALB/c mice. TLRL-2, a synthetic lipopeptide, has demonstrated its efficacy as a potent adjuvant for various vaccines, including those against leishmaniosis [18]. Similarly, TLRL-3, a synthetic analog of double-stranded RNA, has exhibited the ability to enhance a strong T-cell response in mice [16,17]. The HisDTC peptide, both with and without TLRLs, was encapsulated in widely used PLGA nanoparticles known for low cytotoxicity and efficient uptake by dendritic cells [39] as an essential aspect of promoting an effective cellular immune response against *Leishmania* spp. [40]. To assess the efficacy of these peptide-based nanovaccine strategies, we compared them with the LetiFend^®^ vaccine, the only commercially available vaccine against CanL in Europe. Our goal was to evaluate which vaccine strategy elicited the most effective and long-lasting immune response under comparable conditions in the BALB/c model, providing valuable insights into their relative effectiveness.

Studying vaccine-induced immunogenicity prior to infection is a relevant aspect of assessing vaccine efficacy. The DTH response serves as an immunologic parameter, offering insights into cell-mediated immune responses associated with protection or sensibilization to *Leishmania* antigens [41]. To assess the immunogenicity of vaccine nanoformulations, immunized animals were exposed to *L. infantum*-soluble antigens before infection. The DTH reaction observed in mice immunized with HisDTC was significantly stronger and more prolonged compared to the control groups, indicating the development of a Th1-type immune response. In addition, the footpad depth swelling in the LetiFend group was lower than that of HisDTC-vaccinated animals but slightly higher than the control groups. Previous studies in the canine model of CanL revealed that, although the LetiFend^®^ vaccine can induce a heightened DTH response in asymptomatic animals, this response is inconsistent and can vary among individuals [42]. These findings align with previously reported data, suggesting a correlation between the progression of VL and a diminished DTH response, indicating the absence or inadequate cell-mediated immunity [43].

To better analyze vaccine efficacy in controlling parasite multiplication, we examined the parasite burden in target organs, namely the spleen and liver, the primary organs affected by *L. infantum* infection [44]. For the short-term evaluation, subcutaneous immunization with encapsulated HisDTC without TLRLs demonstrated superior efficacy in reducing the parasite load significantly in both the liver and spleen, aligning with prior studies highlighting the protective efficacy of HisDTC against *L. infantum* without any adjuvant [38]. Notably, the HisDTC peptide exhibited better control than LetiFend^®^, a subunit vaccine lacking adjuvants and composed of protein Q, which is a polyprotein resulting from the combination of different antigenic fragments from four different *L. infantum* proteins (Lip2a, Lip2b, P0 and H2a) [43]. HisDTC, comprised specific T cell epitopes with high-MHC class-I- and class-II-binding affinity obtained from four *L. infantum* histones (H2a, H2b, H3, and H4) via bioinformatic analyses, leverages epitope prediction for peptide vaccine development, facilitating the selection of immunogenic epitopes that can promote a more specific and robust Th1 response against *Leishmania* [23,38,45]. The enhanced control capacity of HisDTC in controlling parasite multiplication may also be a consequence of the peptide delivery system. Both groups using the HisDTC peptide were encapsulated in PLGA NPs of around 250 nm in size. Previous research indicates that these polymeric nanoparticles are more efficiently recognized by antigen-presenting cells (APCs) when they are smaller than 500 nm [22]. Additionally, PLGA NPs not only shield the antigen from degradation and enzymatic activity but also offer sustained and prolonged antigen exposure via APCs, facilitating more efficient antigen uptake and presentation, resulting in stronger and more durable responses [46]. In accordance with this, our findings demonstrated the protective capacity in terms of the parasite load sustained over the long-term in HisDTC-immunized animals, a crucial aspect in *Leishmania* vaccine development. Even though parasite multiplication control is achieved in the vaccinated HisDTC group, total parasite clearance is not reached. Nevertheless, in parasitic vaccine development, rather than trying to achieve the complete clearance of the parasite, the goal should be minimizing or eliminating the pathological consequence of infections along with preventing disease transmission [47].

To further investigate the immunological parameters underlying the protective capacity of HisDTC against *Leishmania*, splenocytes and serum were collected from vaccinated and infected mice. The balance of Th1/Th2 immune responses, crucial for controlling *Leishmania* infections, was initially assessed. Cytokines, pivotal in T helper cells’ function regulation, play a role in this balance, with increased production of Th1-associated cytokines, which are essential for infection control, while Th2-related cytokines, such as IL-10, are linked to susceptibility against *L. infantum* multiplication [48]. We focused on key pro-inflammatory cytokines, namely IFN-γ and TNF-α, and the anti-inflammatory cytokine IL-10 to comprehend differences in parasite burden in target organs. Cytokine production in the splenocyte/BMDC co-culture supernatant was analyzed for both short-term and long-term evaluations. Throughout the study, animals immunized with the encapsulated HisDTC peptide, either alone or with TLRLs, consistently induced higher amounts of both IFN-γ and TNF-α compared to other groups, alongside the lower production of the Th2-associated cytokine, IL-10. This pattern aligns with earlier findings for HisDTC, whether used alone or with saponin as an adjuvant in the murine model of VL [38]. No significant differences were observed between short- and long-term evaluation results or when comparing the HisDTC encapsulated peptide alone and in combination with TLRLs. TLRs are specialized in pathogen recognition, activating specific signaling pathways impacting pro-inflammatory and anti-inflammatory cytokine production, which is crucial for protection against *Leishmania* infection [49]. Despite the potential of TLR-based adjuvants to enhance vaccine efficacy [11,50], our results indicate that careful consideration is required due to potential complexities in their effects on the innate and adaptive immunity design [11,50]. Notably, both HisDTC-immunized groups exhibited a pattern that suggests a polarization toward the Th1-type immune response, correlating with the improved control of parasite multiplication in target organs. This Th1-type immune response bias was further supported by the Th1/Th2 characteristic cytokines ratio, highlighting the capacity of both vaccinated groups to induce Th1 predominance in both the short- and long-term evaluation.

In the context of cytokine production, the pivotal role of T CD4^+^ Th1 cells in controlling *L. infantum* infections is well-established. These cells act as mediators by activating dendritic cells, subsequently inducing macrophage activation leading to parasite elimination [51,52,53]. Likewise, CD8^+^-T cell responses have been recognized for their central role in conferring resistance against *Leishmania* spp. This immune response bias not only occurs during initial infection stages but also significantly contributes to resistance upon subsequent reinfection. CD8^+^ T cells can exert cytotoxic activity, killing parasitized macrophages or synthesizing IFN-γ, activating macrophages to enhance their parasite-killing activities, or both [54,55,56]. To ascertain the involvement of T CD4^+^ and CD8^+^ cells in the observed protection against *L. infantum* in HisDTC-vaccinated groups, we evaluated IFN-γ and IL-10 intracellular cytokine production in these cell populations. Our results demonstrated a direct correlation between IFN-γ-producing T CD8^+^ cells and the lowest parasite burden. This aligns with previous studies highlighting the importance of IFN-γ production by T CD8^+^ lymphocytes in protection against *L. infantum* infection [57]. Regarding the percentage of T CD8^+^ T cells producing IL-10 cytokine, several studies support the idea that the absence of IL-10 is correlated with a protective capacity against *L. infantum* infection in mice [58], humans [59], and canids [60]. In line with these findings, HisDTC-immunized animals showed a significantly lower percentage of IL-10^+^-producing CD8^+^ T cells and a preferential secretion of IFN-γ over IL-10 compared to the control and LetiFend groups. This suggests the crucial role of IL-10 in *L. infantum* protection.

One of the key aspects in assessing vaccine efficacy lies in its capacity to elicit and sustain a long-lasting immunological memory response. Effector T lymphocytes, responsible for the initial specific immune response against different pathogens, are complemented by memory T lymphocytes, offering a long-lasting immunity and a rapid response upon re-exposure [61]. In the case of *Leishmania* vaccine development, central memory T (T_CM_) lymphocytes are especially noteworthy, playing a pivotal role in establishing and upholding durable immunity, even in the absence of parasites [62]. Nanoparticle delivery systems and TLRL adjuvants are instrumental in fostering the generation of memory T cells and long-lasting immunity against *Leishmania* [18,36,63]. Their ability to activate antigen-presenting cells in a controlled and sustained manner enhances their value in this context [64]. Our results underscore this, demonstrating that immunization with HisDTC-PLGA formulations led to an increase in the differentiation of both central memory T CD4^+^ and CD8^+^ cells in the spleens of immunized and infected mice. This is evident in the elevated percentage of the T memory cell subpopulation in the long-term evaluation compared to the short-term assessment. The sustained presence of these memory cell subpopulations over an extended period reinforced the notion that mice immunized with encapsulated HisDTC possess the capability to generate robust and enduring T-cell responses upon encountering the parasite with heightened protection. Contrasting the observed reduction in the parasite load in target organs of LetiFend-vaccinated animals, for the short-term evaluation, the LetiFend^®^ vaccine fails to induce a robust and enduring memory T cell response. This is evident from the lack of impact on parasite loads and cytokine production during long-term vaccination (fourteen weeks after vaccination).

Regarding humoral responses, our results reveal that while encapsulated HisDTC did not induce significantly elevated IgG2a production in vaccinated animals, it did reduce IgG1 production compared to the control groups. Interestingly, immunization with HisDTC alone enhanced a higher IgG2a production over IgG1, highlighting the peptide’s role in upregulating Th1-type responses, which correlated with a reduction in parasite burden. It is important to mention the discrepancy from previous results where the HisDTC peptide, administered alone or in combination with saponin as an adjuvant, induced a higher production of IgG2a subclass antibodies [38]. This variance may be explained by the use of PLGA nanoparticles, which, despite inducing a Th1 bias [65], did not result in a higher production of the IgG2a subclass immunoglobulin. Our findings concerning LetiFend groups align with previously described data, affirming the capacity of the LetiFend^®^ vaccine to induce robust IgG2a subclass anti-SLA antibodies in immunized and infected animals [42].

Furthermore, the balance of Th1 immune cell responses associated with a resistant phenotype that allows the control of the disease has been associated with the activation of antigen-presenting cells and the modification of the arginine metabolism. This modification leads to a higher production of NO to the detriment of the activation of the arginase enzyme [66]. In this regard, it can be observed how vaccination strategies capable of inducing better control of parasite multiplication (HisDTC-based vaccines) were the ones capable of inducing the robust production of Th1-characteristic cytokines. IFN-γ can modulate L-arginine metabolism, leading to the control of disease progression. These data are in correlation with previously published findings where the HisDTC peptide, both alone and in combination with saponin, demonstrated the ability to induce parasite control in infected animals due to arginine metabolism modulation [38]. On the other hand, LetiFend and control groups produced higher amounts of IL-10 cytokine, leading to lower iNOS activity, thereby allowing parasite multiplication in target organs as evidenced by parasite loads.

Despite the considerable interest in TLR agonists, specifically TLRL2 and TLRL3, as potential adjuvants in vaccine development, our study revealed a lack of efficacy for these agonists. No significant differences were observed between the groups administered with HisDTC-TLRLs-PLGA and HisDTC-PLGA, indicating that the use of TLR2 (Pam3CSK4) and TLR3 (Poly(I:C)) agonists together did not enhance the synthesis of Th1-type cytokines, including IFN-γ and TNF-α. This finding is unexpected, given the interest in TLR agonists for their capacity to promote a Th1 profile immune response. The role of TLR agonists in *L. infantum* infection is relatively understudied, with most existing data based on *L. major* animal models [67,68,69]. Our results indicate that the combined administration of TLR2 and TLR3 agonists does not significantly impact Th1-type cytokine synthesis. However, there is some immune modulation, reflected in the lower concentration of the IL-10 cytokine both extracellularly and intracellularly in CD8^+^ T cells, particularly during long-term evaluations. This modulation may account for the slight reduction in parasite loads observed in the TLRL-PLGA group compared to the PLGA control group for the long-term evaluation, highlighting the potential contribution of IL-10’s reduction to the successful reduction in parasite burden, as previously described [48,59]. Collectively, our data suggest that the simultaneous administration of TLR2 and TLR3 agonists does not enhance protection against *L. infantum* infection or the immunogenicity induced by the HisDTC peptide. No significant differences were observed in parasite burden, memory T cells, or IgG titers between groups vaccinated with the peptide with or without TLR2/3 agonists. These results are in accordance with a previous study demonstrating that a TLR2 agonist is unable to contribute to protection against *L. infantum* infection in an in vivo murine model [70]. Further investigation of alternative TLR agonists or combinations is warranted in the search for suitable vaccine adjuvants against *L. infantum*.

The results presented in this study, combined with previously reported data, strongly indicate that the multi-epitope HisDTC is a promising tool in combating CanL. Moreover, the use of the HisDTC peptide encapsulated in PLGA nanoparticles, and w/o the presence of adjuvants, enhances a resistant and long-lasting T cell immune response characterized by robust DTH responses, high pro-inflammatory cytokines levels, and central memory T cells. These immune responses correlate with a reduction in the parasite load in the spleen and liver.

## 5. Conclusions

The present study shows the robust and enduring immunoprophylactic efficacy of the chimeric multi-epitope peptide HisDTC encapsulated in PLGA nanoparticles against *L. infantum* infection in the BALB/c murine model. This efficacy is characterized by the establishment of a Th1-type immune response in terms of the vaccine-induced delayed-type hypersensitivity response, the production of IFN-γ and TNF-α, and low IgG1 production. Notably, the activation of IFN-γ-producing T CD8^+^ cells emerged as a pivotal factor in controlling parasite multiplication and promoting the expansion of central memory T cells, contributing to long-lasting immunity. Our study highlights the importance of conducting both short- and long-term evaluation experiments in order to analyze the establishment of a durable and specific immune response, a crucial requirement for the continued exploration of any vaccine candidate. Additionally, the novel use of HisDTC-encapsulated w/o TLR2 and TLR3 agonists demonstrated no significant differences among the vaccinated animals, prompting questions about the efficacy of TLR2 and TLR3 agonists as effective adjuvants for vaccine development against *L. infantum*. These findings open avenues for further research in the field of TLRLs used as adjuvants, considering their potential variability when employed with different delivery systems or against different pathogens, as observed in our study. This study underscores the promising prospects of the HisDTC nanovaccine as a preventive strategy against CanL, emphasizing the importance of PLGA nanoparticles as a promising choice for delivering antigens when strong and lasting T-cell responses. However, for practical application, further studies are imperative to validate the safety and efficacy of this vaccination strategy in dogs, the primary target for protection against this complex infectious disease. Consequently, this study establishes a robust foundation for future research in the ongoing quest to effectively combat CanL.

## 6. Patents

The HisDTC peptide is covered by a patent (EP4163303A1). This patent is the property of the Universidad Complutense de Madrid, Spain.

## Data Availability

The raw data supporting the conclusions of this article will be made available by the authors on request.

## References

[1] Morales-Yuste M., Martín-Sánchez J., Corpas-Lopez V. (2022). Canine Leishmaniasis: Update on Epidemiology, Diagnosis, Treatment, and Prevention. Vet. Sci..

[2] Rocha R., Pereira A., Maia C. (2023). A global perspective on non-autochthonous canine and feline Leishmania infection and leishmaniosis in the 21st century. Acta Trop..

[3] Dantas-Torres F., Solano-Gallego L., Baneth G., Ribeiro V.M., de Paiva-Cavalcanti M., Otranto D. (2012). Canine leishmaniosis in the Old and New Worlds: Unveiled similarities and differences. Trends Parasitol..

[4] WHO Leishmaniasis. https://www.who.int/es/news-room/fact-sheets/detail/leishmaniasis.

[5] Manna L., Reale S., Vitale F., Picillo E., Pavone L.M., Gravino A.E. (2008). Real-time PCR assay in Leishmania-infected dogs treated with meglumine antimoniate and allopurinol. Vet. J..

[6] Segarra S., Miro G., Montoya A., Pardo-Marin L., Boque N., Ferrer L., Ceron J. (2017). Randomized, allopurinol-controlled trial of the effects of dietary nucleotides and active hexose correlated compound in the treatment of canine leishmaniosis. Vet. Parasitol..

[7] Agency E.M. Positive opinion of the Committee for veterinary Medicinal Products for Neoleish. https://www.ema.europa.eu/en/medicines/veterinary/summaries-opinion/neoleish.

[8] Velez R., Gallego M. (2020). Commercially approved vaccines for canine leishmaniosis: A review of available data on their safety and efficacy. Trop. Med. Int. Health.

[9] Calzetta L., Pistocchini E., Ritondo B.L., Roncada P., Palma E., di Cave D., Mattei M., Britti D. (2020). Immunoprophylaxis pharmacotherapy against canine leishmaniosis: A systematic review and meta-analysis on the efficacy of vaccines approved in European Union. Vaccine.

[10] De Brito R.C.F., Cardoso J.M.O., Reis L.E.S., Vieira J.F., Mathias F.A.S., Roatt B.M., Aguiar-Soares R., Ruiz J.C., Resende D.M., Reis A.B. (2018). Peptide Vaccines for Leishmaniasis. Front. Immunol..

[11] Yang J.X., Tseng J.C., Yu G.Y., Luo Y., Huang C.F., Hong Y.R., Chuang T.H. (2022). Recent Advances in the Development of Toll-like Receptor Agonist-Based Vaccine Adjuvants for Infectious Diseases. Pharmaceutics.

[12] Schaut R.G., Grinnage-Pulley T.L., Esch K.J., Toepp A.J., Duthie M.S., Howard R.F., Reed S.G., Petersen C.A. (2016). Recovery of antigen-specific T cell responses from dogs infected with Leishmania (L.) infantum by use of vaccine associated TLR-agonist adjuvant. Vaccine.

[13] Garcon N., Morel S., Didierlaurent A., Descamps D., Wettendorff M., Van Mechelen M. (2011). Development of an AS04-adjuvanted HPV vaccine with the adjuvant system approach. BioDrugs.

[14] Garçon N., Van Mechelen M. (2011). Recent clinical experience with vaccines using MPL- and QS-21-containing adjuvant systems. Expert. Rev. Vaccines.

[15] Didierlaurent A.M., Laupèze B., Di Pasquale A., Hergli N., Collignon C., Garçon N. (2017). Adjuvant system AS01: Helping to overcome the challenges of modern vaccines. Expert. Rev. Vaccines.

[16] Celis E. (2007). Toll-like receptor ligands energize peptide vaccines through multiple paths. Cancer Res..

[17] Fransen F., Boog C.J., van Putten J.P., van der Ley P. (2007). Agonists of Toll-like receptors 3, 4, 7, and 9 are candidates for use as adjuvants in an outer membrane vaccine against Neisseria meningitidis serogroup B. Infect. Immun..

[18] Jayakumar A., Castilho T.M., Park E., Goldsmith-Pestana K., Blackwell J.M., McMahon-Pratt D. (2011). TLR1/2 activation during heterologous prime-boost vaccination (DNA-MVA) enhances CD8+ T Cell responses providing protection against Leishmania (Viannia). PLoS Negl. Trop. Dis..

[19] Thomas S.J., Moreira E.D., Kitchin N., Absalon J., Gurtman A., Lockhart S., Perez J.L., Pérez Marc G., Polack F.P., Zerbini C. (2021). Safety and Efficacy of the BNT162b2 mRNA COVID-19 Vaccine through 6 Months. N. Engl. J. Med..

[20] Prasanna P., Kumar P., Kumar S., Rajana V.K., Kant V., Prasad S.R., Mohan U., Ravichandiran V., Mandal D. (2021). Current status of nanoscale drug delivery and the future of nano-vaccine development for leishmaniasis—A review. Biomed. Pharmacother..

[21] Arevalo C.P., Bolton M.J., Le Sage V., Ye N., Furey C., Muramatsu H., Alameh M.G., Pardi N., Drapeau E.M., Parkhouse K. (2022). A multivalent nucleoside-modified mRNA vaccine against all known influenza virus subtypes. Science.

[22] Silva A.L., Rosalia R.A., Varypataki E., Sibuea S., Ossendorp F., Jiskoot W. (2015). Poly-(lactic-co-glycolic-acid)-based particulate vaccines: Particle uptake by dendritic cells is a key parameter for immune activation. Vaccine.

[23] Martínez-Rodrigo A., Mas A., Álvarez-Campos D., Orden J.A., Domínguez-Bernal G., Carrión J. (2020). Epitope Selection for Fighting Visceral Leishmaniosis: Not All Peptides Function the Same Way. Vaccines.

[24] Carrion J., Abengozar M.A., Fernandez-Reyes M., Sanchez-Martin C., Rial E., Dominguez-Bernal G., Gonzalez-Barroso M.M. (2013). UCP2 deficiency helps to restrict the pathogenesis of experimental cutaneous and visceral leishmaniosis in mice. PLoS Negl. Trop. Dis..

[25] Moreira D., Santarém N., Loureiro I., Tavares J., Silva A.M., Amorim A.M., Ouaissi A., Cordeiro-da-Silva A., Silvestre R. (2012). Impact of continuous axenic cultivation in Leishmania infantum virulence. PLoS Negl. Trop. Dis..

[26] Scott P., Pearce E., Natovitz P., Sher A. (1987). Vaccination against cutaneous leishmaniasis in a murine model. I. Induction of protective immunity with a soluble extract of promastigotes. J. Immunol..

[27] Buffet P.A., Sulahian A., Garin Y.J., Nassar N., Derouin F. (1995). Culture microtitration: A sensitive method for quantifying Leishmania infantum in tissues of infected mice. Antimicrob. Agents Chemother..

[28] Lutz M.B., Kukutsch N., Ogilvie A.L., Rössner S., Koch F., Romani N., Schuler G. (1999). An advanced culture method for generating large quantities of highly pure dendritic cells from mouse bone marrow. J. Immunol. Methods.

[29] Ding A.H., Nathan C.F., Stuehr D.J. (1988). Release of reactive nitrogen intermediates and reactive oxygen intermediates from mouse peritoneal macrophages. Comparison of activating cytokines and evidence for independent production. J. Immunol..

[30] Garrido V.V., Dulgerian L.R., Stempin C.C., Cerbán F.M. (2011). The increase in mannose receptor recycling favors arginase induction and Trypanosoma cruzi survival in macrophages. Int. J. Biol. Sci..

[31] Dominguez-Bernal G., Martinez-Rodrigo A., Mas A., Blanco M.M., Orden J.A., De La Fuente R., Carrion J. (2017). Alternative strategy for visceral leishmaniosis control: HisAK70-Salmonella Choleraesuis-pulsed dendritic cells. Comp. Immunol. Microbiol. Infect. Dis..

[32] Scott P. (2004). Immunoparasitology. Immunol. Rev..

[33] Wanasen N., Soong L. (2008). L-arginine metabolism and its impact on host immunity against Leishmania infection. Immunol. Res..

[34] Giunchetti R.C., Silveira P., Resende L.A., Leite J.C., Melo-Junior O.A.O., Rodrigues-Alves M.L., Costa L.M., Lair D.F., Chaves V.R., Soares I.D.S. (2019). Canine visceral leishmaniasis biomarkers and their employment in vaccines. Vet. Parasitol..

[35] Sabur A., Bhowmick S., Chhajer R., Ejazi S.A., Didwania N., Asad M., Bhattacharyya A., Sinha U., Ali N. (2018). Liposomal Elongation Factor-1α Triggers Effector CD4 and CD8 T Cells for Induction of Long-Lasting Protective Immunity against Visceral Leishmaniasis. Front. Immunol..

[36] Agallou M., Margaroni M., Kotsakis S.D., Karagouni E. (2020). A Canine-Directed Chimeric Multi-Epitope Vaccine Induced Protective Immune Responses in BALB/c Mice Infected with Leishmania infantum. Vaccines.

[37] Agallou M., Margaroni M., Tsanaktsidou E., Badounas F., Kammona O., Kiparissides C., Karagouni E. (2023). A liposomal vaccine promotes strong adaptive immune responses via dendritic cell activation in draining lymph nodes. J. Control. Release Off. J. Control. Release Soc..

[38] Mas A., Hurtado-Morillas C., Martínez-Rodrigo A., Orden J.A., de la Fuente R., Domínguez-Bernal G., Carrión J. (2023). A Tailored Approach to Leishmaniases Vaccination: Comparative Evaluation of the Efficacy and Cross-Protection Capacity of DNA vs. Peptide-Based Vaccines in a Murine Model. Int. J. Mol. Sci..

[39] Margaroni M., Agallou M., Kontonikola K., Karidi K., Kammona O., Kiparissides C., Gaitanaki C., Karagouni E. (2016). PLGA nanoparticles modified with a TNFα mimicking peptide, soluble Leishmania antigens and MPLA induce T cell priming in vitro via dendritic cell functional differentiation. Eur. J. Pharm. Biopharm..

[40] Soong L. (2008). Modulation of dendritic cell function by Leishmania parasites. J. Immunol..

[41] De Rossell R.A., Bray R.S., Alexander J. (1987). The correlation between delayed hypersensitivity, lymphocyte activation and protective immunity in experimental murine leishmaniasis. Parasite Immunol..

[42] Carcelén J., Iniesta V., Fernández-Cotrina J., Serrano F., Parejo J.C., Corraliza I., Gallardo-Soler A., Marañón F., Soto M., Alonso C. (2009). The chimerical multi-component Q protein from Leishmania in the absence of adjuvant protects dogs against an experimental Leishmania infantum infection. Vaccine.

[43] Pacheco-Fernandez T., Volpedo G., Gannavaram S., Bhattacharya P., Dey R., Satoskar A., Matlashewski G., Nakhasi H.L. (2021). Revival of Leishmanization and Leishmanin. Front. Cell Infect. Microbiol..

[44] Loeuillet C., Bañuls A.L., Hide M. (2016). Study of Leishmania pathogenesis in mice: Experimental considerations. Parasit. Vectors.

[45] Agallou M., Athanasiou E., Koutsoni O., Dotsika E., Karagouni E. (2014). Experimental Validation of Multi-Epitope Peptides Including Promising MHC Class I- and II-Restricted Epitopes of Four Known Leishmania infantum Proteins. Front. Immunol..

[46] Cruz L.J., Tacken P.J., Eich C., Rueda F., Torensma R., Figdor C.G. (2017). Controlled release of antigen and Toll-like receptor ligands from PLGA nanoparticles enhances immunogenicity. Nanomedicine.

[47] Donnelly S. (2022). The immunology of parasite infections: Grand challenges. Front. Parasitol..

[48] Kaye P.M., Cruz I., Picado A., Van Bocxlaer K., Croft S.L. (2020). Leishmaniasis immunopathology-impact on design and use of vaccines, diagnostics and drugs. Semin. Immunopathol..

[49] Martinez-Orellana P., Quirola-Amores P., Montserrat-Sangra S., Ordeix L., Llull J., Alvarez-Fernandez A., Solano-Gallego L. (2017). The inflammatory cytokine effect of Pam3CSK4 TLR2 agonist alone or in combination with Leishmania infantum antigen on ex-vivo whole blood from sick and resistant dogs. Parasit. Vectors.

[50] Lu B.L., Williams G.M., Brimble M.A. (2020). TLR2 agonists and their structure-activity relationships. Org. Biomol. Chem..

[51] Romano A., Doria N.A., Mendez J., Sacks D.L., Peters N.C. (2015). Cutaneous Infection with Leishmania major Mediates Heterologous Protection against Visceral Infection with Leishmania infantum. J. Immunol..

[52] Coler R.N., Goto Y., Bogatzki L., Raman V., Reed S.G. (2007). Leish-111f, a recombinant polyprotein vaccine that protects against visceral Leishmaniasis by elicitation of CD4+ T cells. Infect. Immun..

[53] Hohman L.S., Peters N.C. (2019). CD4(+) T Cell-Mediated Immunity against the Phagosomal Pathogen Leishmania: Implications for Vaccination. Trends Parasitol..

[54] Stäger S., Rafati S. (2012). CD8(+) T cells in leishmania infections: Friends or foes?. Front. Immunol..

[55] Basu R., Bhaumik S., Haldar A.K., Naskar K., De T., Dana S.K., Walden P., Roy S. (2007). Hybrid cell vaccination resolves Leishmania donovani infection by eliciting a strong CD8+ cytotoxic T-lymphocyte response with concomitant suppression of interleukin-10 (IL-10) but not IL-4 or IL-13. Infect. Immun..

[56] Polley R., Stager S., Prickett S., Maroof A., Zubairi S., Smith D.F., Kaye P.M. (2006). Adoptive immunotherapy against experimental visceral leishmaniasis with CD8+ T cells requires the presence of cognate antigen. Infect. Immun..

[57] Athanasiou E., Agallou M., Tastsoglou S., Kammona O., Hatzigeorgiou A., Kiparissides C., Karagouni E. (2017). A Poly(Lactic-co-Glycolic) Acid Nanovaccine Based on Chimeric Peptides from Different Leishmania infantum Proteins Induces Dendritic Cells Maturation and Promotes Peptide-Specific IFNγ-Producing CD8(+) T Cells Essential for the Protection against Experimental Visceral Leishmaniasis. Front. Immunol..

[58] Murphy M.L., Wille U., Villegas E.N., Hunter C.A., Farrell J.P. (2001). IL-10 mediates susceptibility to Leishmania donovani infection. Eur. J. Immunol..

[59] Vouldoukis I., Bécherel P.A., Riveros-Moreno V., Arock M., da Silva O., Debré P., Mazier D., Mossalayi M.D. (1997). Interleukin-10 and interleukin-4 inhibit intracellular killing of Leishmania infantum and Leishmania major by human macrophages by decreasing nitric oxide generation. Eur. J. Immunol..

[60] de Oliveira Cardoso J.M., de Brito R.C.F., Costa A.F.P., Siqueira Mathias F.A., Soares Reis L.E., Vieira J.F.P., de Oliveira Aguiar Soares R.D., Reis A.B., Roatt B.M. (2021). IL-10 receptor blockade controls the in vitro infectivity of Leishmania infantum and promotes a Th1 activation in PBMC of dogs with visceral leishmaniasis. Mol. Immunol..

[61] Ojdana D., Safiejko K., Lipska A., Radziwon P., Dadan J., Tryniszewska E. (2008). Effector and memory CD4+ and CD8+ T cells in the chronic infection process. Folia Histochem. Cytobiol..

[62] Zaph C., Uzonna J., Beverley S.M., Scott P. (2004). Central memory T cells mediate long-term immunity to Leishmania major in the absence of persistent parasites. Nat. Med..

[63] Agallou M., Margaroni M., Karagouni E. (2023). Intramuscular Immunization with a Liposomal Multi-Epitope Chimeric Protein Induces Strong Cellular Immune Responses against Visceral Leishmaniasis. Vaccines.

[64] Demento S.L., Cui W., Criscione J.M., Stern E., Tulipan J., Kaech S.M., Fahmy T.M. (2012). Role of sustained antigen release from nanoparticle vaccines in shaping the T cell memory phenotype. Biomaterials.

[65] Gu P., Wusiman A., Wang S., Zhang Y., Liu Z., Hu Y., Liu J., Wang D. (2019). Polyethylenimine-coated PLGA nanoparticles-encapsulated Angelica sinensis polysaccharide as an adjuvant to enhance immune responses. Carbohydr. Polym..

[66] Martinez-Rodrigo A., D S.D., Ribeiro P.A.F., Roatt B.M., Mas A., Carrion J., Coelho E.A.F., Dominguez-Bernal G. (2019). Immunization with the HisAK70 DNA Vaccine Induces Resistance against Leishmania Amazonensis Infection in BALB/c Mice. Vaccines.

[67] Huang L., Hinchman M., Mendez S. (2015). Coinjection with TLR2 agonist Pam3CSK4 reduces the pathology of leishmanization in mice. PLoS Negl. Trop. Dis..

[68] Halliday A., Turner J.D., Guimarães A., Bates P.A., Taylor M.J. (2016). The TLR2/6 ligand PAM2CSK4 is a Th2 polarizing adjuvant in Leishmania major and Brugia malayi murine vaccine models. Parasit. Vectors.

[69] Pandey S.P., Chandel H.S., Srivastava S., Selvaraj S., Jha M.K., Shukla D., Ebensen T., Guzman C.A., Saha B. (2014). Pegylated bisacycloxypropylcysteine, a diacylated lipopeptide ligand of TLR6, plays a host-protective role against experimental Leishmania major infection. J. Immunol..

[70] Salgado C.L., Dias E.L., Stringari L.L., Covre L.P., Dietze R., Lima Pereira F.E., de Matos Guedes H.L., Rossi-Bergmann B., Gomes D.C.O. (2019). Pam3CSK4 adjuvant given intranasally boosts anti-Leishmania immunogenicity but not protective immune responses conferred by LaAg vaccine against visceral leishmaniasis. Microbes Infect..

